# Minimum Variance Distortionless Response Beamformer with Enhanced Nulling Level Control via Dynamic Mutated Artificial Immune System

**DOI:** 10.1155/2014/164053

**Published:** 2014-06-05

**Authors:** Tiong Sieh Kiong, S. Balasem Salem, Johnny Koh Siaw Paw, K. Prajindra Sankar, Soodabeh Darzi

**Affiliations:** ^1^Power Engineering Center, College of Engineering, Universiti Tenaga Nasional, Kajang, Selangor, Malaysia; ^2^Center of System and Machine Intelligence, College of Engineering, Universiti Tenaga Nasional, Kajang, Selangor, Malaysia; ^3^Department of Electrical, Electronic & Systems Engineering, Universiti Kebangsaan Malaysia, Bangi, Selangor, Malaysia

## Abstract

In smart antenna applications, the adaptive beamforming technique is used to cancel interfering signals (placing nulls) and produce or steer a strong beam toward the target signal according to the calculated weight vectors. Minimum variance distortionless response (MVDR) beamforming is capable of determining the weight vectors for beam steering; however, its nulling level on the interference sources remains unsatisfactory. Beamforming can be considered as an optimization problem, such that optimal weight vector should be obtained through computation. Hence, in this paper, a new dynamic mutated artificial immune system (DM-AIS) is proposed to enhance MVDR beamforming for controlling the null steering of interference and increase the signal to interference noise ratio (SINR) for wanted signals.

## 1. Introduction

The evolution of adaptive beamforming was initiated in military and aerospace applications through the employment of electronically steered antennas based on phased-array technology. Typical applications include long-range surveillance radar, active jammer rejection, and multibeam antennas for space communications [1]. The same antenna array techniques were then assumed suitable for mobile radio communication to solve multipath fading and the cochannel interference problem. A partially adaptive antenna array technology, known as the intermediate frequency side lobe canceller (SLC), was invented by Howells in the late 1950s [2]. This technology incorporates the capability of automatic interference nulling. However, SLC was not fully adaptive because the main beam has a fixed pattern, and the auxiliary array contains only a few controlled elements. This simple adaptive antenna facilitated the development of fully adaptive array by Howells' coworker, Applebaum, in 1965. The algorithm, commonly known as the Howells-Applebaum algorithm, was developed to maximize the signal-to-noise ratio (SNR) at the output of the beamformer. At the same time, in 1960, another independent research group led by Windrow invented another adaptive array approach based on linear covariance minimum variance (LCMV) [3]. This algorithm, later known as the Windrow-Hoff LMS algorithm, was developed based on the minimum mean square error (MMSE) criterion for the automatic adjustment of array weights. This algorithm is well known for its simplicity but only achieves satisfactory performance under specific operational conditions. The major drawback of this algorithm is its low convergence rate, which refers to the speed by which the mean of the estimated weights approaches the optimal value, thereby making it unsuitable for certain applications.

In 1969, Capon introduced another adaptive beamformer known as minimum variance distortionless response (MVDR), which is a technique capable of resolving signals separated by a fraction of antenna beam width. This optimum beamformer, also known as the LCMV beamformer, requires only the knowledge of the desired signal direction of arrival (DOA) to maximize the SNR. Another important contribution was by Reed, Mallett, and Brennen in 1974. They introduced a fast convergence algorithm known as the sample matrix inversion (SMI) technique, which overcame the problem of slow convergence faced by the LMS algorithm. One of the most important factors in smart antenna processes is beamforming, which refers to the allocation of signals in particular positions and phase angles for each antenna for the corresponding angle of the system [4].

Beamforming technology is a key technique in nulling antenna. By performing some processes with the received array signals, such as weighting and summation, beamforming can help the antenna realize many advanced functions, such as beam shaping, beam scanning, and beam nulling [5]. Reception beamforming is independently achieved at each receiver; however, the transmitter in transmit beamforming has to consider all receivers to optimize the beamformer output [6, 7].

One of the beamforming algorithm used in smart antenna is MVDR beamforming, which can calculate the weight vector to determine the desired signal from the interference. Moreover, MDVR maximizes the sensitivity in one direction only [8]. The MVDR beamformer, also known as Capon beamformer, minimizes the output power of the beamformer under a single linear constraint on the response of the array toward the desired signal. The idea of combining multiple inputs in a statistically optimum manner under the constraint of no signal distortion can be attributed to Darlington. Several researchers developed beamformers, in which additional linear constraints are imposed (e.g., Er and Cantoni) [9, 10].

Many approaches proposed the use of a mathematical model to improve the robustness of the MVDR beamformer, as presented in [11, 12]. Research on the artificial immune system (AIS) and its application has become increasingly important in the field of intelligent information systems [13–15]. A new optimization technique was presented for the design of linear antenna arrays. The proposed technique was based on a novel variant of particle swarm optimization (PSO) called Boolean PSO with adaptive velocity mutation. The antenna arrays were optimized based on the requirements for maximizing the power gain at a desired direction and minimizing the side lobe level of the radiation pattern [16]. A complex-valued genetic algorithm for the optimization of beamforming in linear array antennas was proposed. The algorithm was proven to enhance searching efficiency significantly while effectively avoiding premature convergence. Numerical results were presented to illustrate the advantages of the proposed technique over conventional pattern synthesis methods [17].

In this paper, the main goal is to design a beamforming method based on MVDR in corporation with new dynamic mutated artificial immune system (DM-AIS) algorithm in order to enhance the null level at interference sources. By using this method, finding a new mathematical model, changing the filter hardware for signal processing, or changing the design of antenna based on the increased number of elements is no longer necessary. Another reason for proposing the new method is the difficulty in obtaining the optimum value using any normal algorithm without intensification. In this investigation, DM-AIS have been applied in beamforming with uniform linear antenna arrays of 0.5 *λ* spacing between adjacent elements and radiating at a frequency of 2.3 GHz. The rest of this paper is organized as follows.  Section 2 introduces the basics of adaptive beamforming. Sections 3 and 4 summarized a basis to describe the conventional MVDR beamforming and AIS, respectively.  Section 5 shows the incorporation of MVDR with DM-AIS. Simulation results of one user with two interferences and comparison of conventional MVDR with mp-QP MVDR and DM-AIS are reported in Section 6. And finally Section 7 concludes this investigation.

## 2. Background of Adaptive Beamforming

Adaptive beamforming is a technique for receiving a signal of interest (SOI) from specific directions while suppressing the interfering signals adaptively in other directions using an array of sensors. This technique can automatically optimize the array pattern by adjusting the elemental control weights until a prescribed objective function is satisfied. This technique provides a means for separating a desired signal from interfering signals.

**Figure 1:**
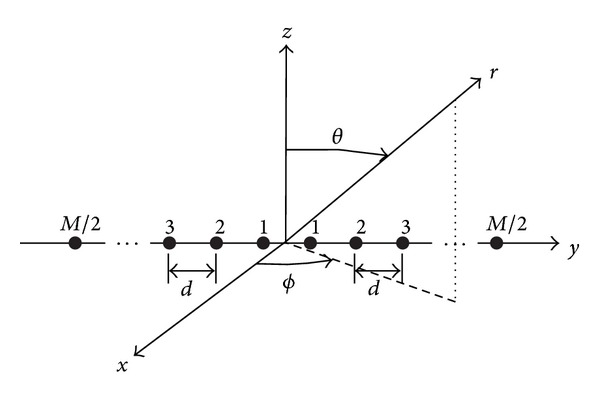
Linear array with elements along the *y*-axis.

Beamforming has numerous applications in radar, sonar, seismology, microphone array speech processing, and, more recently, wireless communications. In particular, the use of antenna arrays, in combination with signal processing algorithms at the base station, offers the possibility of exploiting the spatial dimension to separate multiple cochannel users. This approach provided increased channel capacity and wider area coverage. Array beamforming methods in such systems use the spatial dimension to combat interference, noise, and multipath fading of the desired signal [11]. The outputs of the individual sensors were linearly combined after being scaled with the corresponding weights. This process optimizes the antenna array to achieve maximum gain in the direction of the desired signal and nulls in the direction of interferers. For a beamformer, the output at any time *n*, *y*(*n*) is given by a linear combination of the data at *M* antennas, with *x*(*n*) being the input vector and *w*(*n*) being the weight vector, as shown in Figure 1:

(1) $$\begin{array}{c} y\left(n\right)=w^{H}{\left(n\right)}^{*}\left(n\right). \end{array}$$

Weight vector *W*(*n*) can be defined as follows:

(2) $$\begin{array}{c} w\left(n\right)=\overset{M-1}{\underset{N=0}{\sum }}w_{n},\\ x\left(n\right)=\overset{M-1}{\underset{n=0}{\sum }}X_{n}. \end{array}$$

For any algorithm that evades the matrix inverse operation and uses the immediate gradient vector ∇*J*(*n*) for weight vector upgrading, the weight vector at time *n* + 1 can be written as follows:

(3) $$\begin{array}{c} W\left(n+1\right)=W\left(n\right)+\frac{1}{2}\mu \left[\nabla J\left(n\right)\right], \end{array}$$

where *μ* is the step size parameter, which controls the speed of convergence and lies between 0 and 1. The minimum values of *μ* facilitate the sluggish concurrence and high-quality estimation of the cost function. Comparatively, the huge values of *μ* may direct to a rapid union. However, the constancy over the least value may disappear:

(4) $$\begin{array}{c} 0<\mu <\frac{1}{\lambda }. \end{array}$$

An exact calculation of instantaneous gradient vector ∇*J*(*n*) is not possible because prior information on covariance matrix *R* and cross-correlation vector *p* is needed. Thus, an instantaneous estimate of gradient vector is given by

(5) ∇J(n)=−2p(n)+2R(n)W(n)R(n)=X(n)XH(n),P(n)=d(n)∗X(n).

By substituting values from (5) into (3), the weight vector is derived as follows:

(6) W(n+1)=W(n)+μ[p(n)−R(n)W(n)]=W(n)+μX(n)⌊d∗(n)−X(n)W(n)⌋=W(n)+μXe∗(n).

The desired signal can be defined by the following three equations:

(7) y(n)=wH(n)x(n)e(n)=d(n)·y(n)W(n+1)=W(n)+μX(n)e∗(n).

Numerous algorithms were introduced for the design of an adaptive beamformer [8]. One of the most popular approaches for adaptive beamforming was proposed by Capon [4]. His algorithm leads to an adaptive beamformer with an MVDR. Some constraints, such as the antenna gain being constant in the desired direction, are used to ensure that the desired signals are not filtered out along with the unwanted signals. The MVDR beamformer not only minimizes the array output power but also maintains a distortionless main lobe response toward the desired signal. However, the MVDR beamformer may have an unacceptably low nulling level, which may significantly degrade the performance in the case of unexpected interfering signals. In particular, the performance of MVDR degrades in rapidly moving jammer environments. This degradation occurs because of jammer motion, which may bring jammers out of the sharp notches of the adapted pattern. To achieve high interference suppression and SOI enhancement, an adaptive array must introduce deep and widened nulls in the DOAs of strong interferences while keeping the desired signal distortionless. Thus, the issue of nulling level control is especially important for both deterministic and adaptive arrays [18].

## 3. Conventional MVDR Beamforming

When a beamformer has a constant response in the direction of a useful signal, the LCMV algorithm becomes an MVDR algorithm [19]. The MVDR algorithm is capable of suppressing the interference, but with high value in SNR and low noise. At the same time, the MVDR algorithm depends on the steering vectors, which in turn depend on the incident angle of the received signal from the element of the array antenna. The direction of useful signal must be known and the output power subject to a unity gain constraint in the direction of desired signal must be minimized. The array output is given by

(8) $$\begin{array}{c} y=w^{H}x. \end{array}$$

The output power is as follows:

(9) $$\begin{array}{c} p=\left\{E{\left|y\right|}^{2}\right\}=E\left\{w^{H}xx^{H}w\right\}=w^{H}E\left\{xx^{H}w\right\}=w^{H}R, \end{array}$$

where the *R* covariance matrix should be (*M*, 1) for the received signal *x* and *H* is the hermitian transpose.

The optimum weights are selected to minimize the array output power *P*_MVDR_ while maintaining unity gain in the look direction *a*(*θ*), which is the steering vector of the desired signal. The MVDR adaptive algorithm can be written as follows:

(10) $$\begin{array}{c} \text{mi}{\text{n}}_{w}\left\{w^{H}Rw\right\}\;\text{subject}\;\text{to}\;w^{H}a\left(\theta \right)=1. \end{array}$$

The steering vector *a*(*θ*) is given by

**Figure 2:**
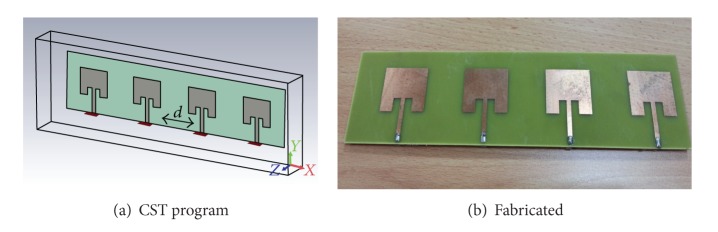
(a, b) Four elements of the linear array.

(11) a(θ)=[1exp⁡{j2πλ(sinθi)d}exp⁡{j2πλ(sinθi)(m−1)d}],

where *d* is the space between the elements of the antenna, *θ*_*i*_ is the desired angle, and *m* is the number of elements, as shown in Figure 2.

The optimization weight vectors can then be acquired using the following formula [20]:

(12) $$\begin{array}{c} W_{\text{MVDR}}=\frac{R^{-1}a\left(\theta \right)}{a^{H}\left(\theta \right)R^{-1}a\left(\theta \right)}. \end{array}$$

These weights are the solution of the optimization problem mentioned at (10) and with the use of four elements of array antenna, four weights as below will be obtained:

(13) $$\begin{array}{c} w_{\text{MVDR}}=\left[\begin{array}{c} w_{1}\\ w_{2}\\ w_{3}\\ w_{4} \end{array}\right]. \end{array}$$

Subsequently, the beamformer weights are selected based on minimum mean value of output power according to the number of users inside the coverage area while maintaining unity response in the desired direction. Nevertheless, the restraint ensures that the signal passes through the beamformer undistorted. Consequently, the output signal power is similar to the look direction source power. The total noise, including interferences and uncorrelated noise, is then reduced by the minimization process. Notably, the minimization of the total output noise, while constantly maintaining the output signal, is the same as maximizing the output SINR. However, for the optimal beamformer to perform as described above and to maximize the SINR by cancelling interferences, the number of interferences must be less than or equal to *M* − 2 because an array with *M* elements has *M* − 1 degrees of freedom and has been utilized by the constraint in the look direction. Given that the MVDR beamformer maximizes sensitivity in one direction only, this beamformer is unsuitable for multipath environments, where the desired signal spreads in all directions [21]. The multipath occurs in non-line-of-sight environments such as populated urban areas, where numeorus scatterers are close to the users and the base station. Thus, the MVDR beamformer may have an unacceptably low nulling level, which may significantly degrade performance in the case of unexpected interfering signals. As a result, the beamforming optimization problem is formulated as a multiparametric quadratic programming (mp-QP) [18].

## 4. Artificial Immune System

The proposed usage of artificial intelligent system as the enhancement method for the adaptive beamforming technique is based on a framework that is built around the concept of reactive artificial immune system (AIS). AIS, which is inspired by theoretical immunology and observed immune functions, is a branch of the metaheuristic algorithm with promising results in the field of optimization. While AIS resembles some other metaheuristics algorithms such as genetic algorithm (GA), except the recombination operator, the former has code simplicity and is low in computational cost. This AIS system is indeed based upon the normal human immune system in the way that it is reactive towards foreign elements. The immune system is highly robust, adaptive, inherently parallel, and self-organized. It has powerful learning and memory capabilities and presents an evolutionary type of response to infectious foreign elements [22, 23].

The main agents of the adaptive immune system are lymphocytes that are are called the B cells which produce antibodies to attack the enemy. Some B cells become “memory cells” which keep molecular records of past invader and minimize the body's response time to an infection. The clonal selection principle is the whole process of antigen recognition, cell proliferation, and differentiation into a memory cell.

The clonal-selection theory proposes that as an antigen enters the immune system certain B cells are selected based on their reaction to this antigen to undergo rapid cloning and expansion. This reaction is often termed the affinity of that B cell (or antibody) for the given antigen. Those B cells are selected, based on their affinity to an antigen, to produce a number of clones to attack or neutralize the invading antigen. Cells that have a higher degree of affinity are allowed to produce more clones. The clonal production develops immune cells that are more adept at recognizing and reacting to the antigen through mutation. B cell offsprings undergo mutation based on an inverse proportionality to their affinity values. Through this process, the affinity of subsequent generations of B cells will have greater reaction to the antigen, and more diversity will also have been added to the system through the wider exploration afforded by the high mutation rates of the cells with lower affinity measures.

The process of a standard clonal selection algorithm can be summarized as follows [24, 25].

(1) Generate a random initial population of antibody *Ab*, given by
  (14) $$\begin{array}{c} P\left(0\right):=\left\{Ab_{1}\left(0\right),\ldots .,Ab_{n}\left(0\right)\right\}. \end{array}$$
(2) Compute the fitness of each *Ab*
  (15) $$\begin{array}{c} P\left(0\right):\left\{f\left(x_{1}\left(0\right),\ldots .,x_{n}\left(0\right)\right)\right\}. \end{array}$$
(3) Generate clones by cloning all cells in the *Ab* population. The amount of clone is given by
  (16) $$\begin{array}{c} N_{C}=\overset{n}{\underset{i=1}{\sum }}\text{round}\left(\frac{\beta •N}{i}\right), \end{array}$$
  where *N*_*C*_ is the total clones generated for each *Ab*, *β* is the multiplying factor, and *N* is the number of *Ab*.
(4) Mutate the clone population to produce a mature clone population with *δ* number of children. The new *Ab* is composed of
  (17) $$\begin{array}{c} P^{\prime }:=\left\{Ab_{1}^{\prime },\ldots .,Ab_{\delta }^{\prime }\right\}. \end{array}$$
(5) Evaluating affinity values of the clones' population is
  (18) $$\begin{array}{c} P^{\prime }:\left\{f\left(x_{1}^{\prime },\ldots .,x_{\delta }^{\prime }\right)\right\}. \end{array}$$
  The next generation of *Ab* is obtained using *G* = *ε* + *δ* selection by choosing the best *ε* individuals out of the *G* population.
(6) Select the best *Ab* to compose the new *Ab* population by
  (19) $$\begin{array}{c} P_{\text{new}}:=s_{G}P^{\prime }. \end{array}$$
(7) Steps 3 to 6 are repeated until a predefined stopping condition is reached.

## 5. MVDR Beamforming Incorporation with New Dynamic Mutated Artificial Immune System

In this paper, AIS with dynamic mutation (DM) was utilized to enhance the null level of the MVDR beamforming technique. In analogy, the adaptive antenna represents the body of an organism, whereas the interference and noise signal sources represent external harmful attacks toward the organism. Hence, the adaptive antenna system will organize its antibody to protect the body of organism from external antigen attacks. The adaptive antenna system will try to optimize through its AIS iteration process to develop deep null at the DOA of the interference sources to achieve the maximum SINR.

In this AIS algorithm, the weight vector *w* will be generated as the system antibody. The algorithm will initiate by generating a population of *N* antibodies, which is represented by weight vectors *W*_*N*_. The number of generated weight vectors depends on the population size *P*_size_. For the first iteration, the first set of weight vectors *W*_1_ is obtained from the computation of the conventional MVDR weight vector. The weight vectors in every antibody contain an *M* number of weight vectors, depending on the number of sensors or antenna elements used, and can be expressed as follows:

(20) w1M=Real{wmvdr}M+Imag{wmvdr}Mw2M=Real{w1M∗rand(M,PSize−1)}+Imag{w1M∗rand(M,PSize−1)}⋮wNM=Real{w(N−1)M∗rand(M,PSize−1)}+Imag{w(N−1)M∗  rand(M,PSize−1)}.

In matrix format, the weight vectors in the population of any iteration can be represented by

(21) $$\begin{array}{c} W_{NM}=\left[\begin{array}{cccc} w_{\text{mvdr}1} & w_{\text{mvdr}2} & w_{\text{mvdr}3}\; & w_{\text{mvdr}4}\\ w_{11} & \;w_{12} & w_{13}\; & w_{14}\\ w_{21} & w_{22}\; & w_{23} & w_{24}\\ . & . & . & .\\ . & . & . & .\\ w_{n1} & w_{n2} & w_{n3} & w_{n4} \end{array}\right], \end{array}$$

where

 
*W*_*NM*_ is weight vectors of total population *N* with *M* sensors in each antenna;
 
*w*_mvdr_ is weight vectors from MVDR beamformer;
 
*M* is number of sensor. In this study, antenna sensor of (4, 1) is used;
 
*P*_size_ is population size;
 
*M* is number of sensor.

Each set of antibodies *W* has amplitude and phase (*A*∀ *θ*) to steer the radiation beam toward its target user and place the deep null toward the interference sources to achieve the optimum SINR. The best weight vector is determined according to the fitness value obtained from fitness function as shown below:

(22) $$\begin{array}{c} \text{Fitness}\_\text{Function}\;\left(\text{FF}\right)=\frac{P_{\text{User}}}{\sum_{n=1}^{N}P_{\text{Inter}\_n}+\text{Noise}}, \end{array}$$

where *P*_User_ = power of target user, *P*_Inter_ = power of interference, and *N* = number of interference sources.

The new candidate population of antibodies (weight vectors) based on the mutation rate depends on the fitness value of *w* in (22), which is given by

(23) $$\begin{array}{c} M_{\text{rate}}=e^{-(\sqrt[{5}]{{\left(Fw_{\text{best}}\right)}^{2}})}, \end{array}$$

where *M*_rate_ = mutation rate and *Fw*_best_ = fitness of best population weight.

The mutation rate of clones is inversely proportional to their antigenic affinity [24, 26]. A higher affinity denotes a smaller mutation rate.

The mutations and clones needed to create the new weight are given by

(24)   W=wbest+Mrate∗(Real{rand(M,PSize−1)}−Imag{rand(M,PSize−1)}).

The *n* antibodies generate *Nc* clones proportional to their affinities. The number of cloned antibodies *Nc* can be computed by

(25) $$\begin{array}{c} \;\;\;\;\;\;N_{C}=\overset{n}{\underset{i=1}{\sum }}\text{round}\frac{B\cdot w}{i}, \end{array}$$

where *B* is a multiplying factor with a value of 1 and *w* is the weight vector.

Each term of this sum corresponds to the clone size of each selected antibody. The clones are then subjected to hyper mutation and receptor editing. To enable the algorithm to find the best solution, a deep null must be achieved to obtain the maximum SINR. Therefore, the DM was introduced to identify the best solution. The selection of the 10 best solutions depends on the maximum SINR value. Thus, from these 10 best solutions, DM-AIS can select three random values from the 10 best weights to achieve the SINR size. When the scaling factor is (0, 1, 2), the best value of the deep null is derived:

(26) $$\begin{array}{c} \;\;w_{DM}=\;w_{1M}+{\text{sf}}^{*}\left(w_{2M}-w_{3M}\right), \end{array}$$

where *W*_1_, *W*_2_, *W*_3_ = random weight select from the best three solutions and sf = scaling factor from (0, 1, 2).

The next step is the clonal operation to obtain the best solution.

Affinity is applied to achieve the maximum power for target and deep null for interference. If this value is the best, it will store and yield the final weight value to stop the calculation. The DM-AIS steps in an adaptive antenna are as follows.

(1) A random initial population of *W* is generated, which is given by
  (27) $$\begin{array}{c} W_{N}\left(i\right):=\left\{W_{1}\left(i\right),\ldots ,W_{n}\left(i\right)\right\}. \end{array}$$
(2) The fitness of each *W* is computed:
  (28) $$\begin{array}{c} P\left(i\right):\left\{f\left(w_{1}\left(i\right),\ldots ,w_{n}\left(i\right)\right)\right\}. \end{array}$$
(3) Clones are generated by cloning all the cells in the *W* population. The amount of clone is obtained using (23).
(4) The clone population is mutated to produce a mature clone population with *i* number of weight. The rate of mutation is given by
  (29) $$\begin{array}{c} M_{\text{rate}}=e^{-(\sqrt[{5}]{{\left(Fw_{\text{best}}\right)}^{2}\;})}. \end{array}$$
  Thus, the new *W* is composed of *W*_*N*_′(*i*) = {*W*_1_′,…, *W*_*i*_′}.
(5) The affinity values of the clone population are expressed as follows:
  (30) $$\begin{array}{c} W_{N}^{\prime }\left(i\right):\left\{f\left(W_{1}^{\prime },\ldots ,W_{i}^{\prime }\right)\right\}. \end{array}$$
  The next generation of *W* is obtained using (20). The best *W*_best_ is selected to compose the new *W*_new_ population as follows:
  (31) $$\begin{array}{c} W_{\text{new}}=W_{\text{best}}\;W_{N}^{\prime }\left(i\right). \end{array}$$
(6) The 10 best weight vectors are selected depending on the selection assumption (*P*_size_) to identify the best number of the weight.
(7) The vectors are mutated by selecting three random weights from the 10 best weights using (26).
(8) Steps 3 to 6 are repeated until a predefined stopping condition is reached.

*Remarks*. Similar to weight vector optimization techniques based on the support vector regression (SVR-AS) [27] and mp-QP MVDR [18], the proposed DM-AIS MVDR technique is also effective in finding the optimal weight vector for an antenna array under specific conditions. The differences among the methodologies are as follows.

1. The proposed DM-AIS MVDR determines the optimal weight vector of an antenna array through its cloning and mutation process to fine-tune the weight vector toward a radiation pattern that can generate deep null toward the interference source, while maintaining high gain at the target users' directions for an instantaneous scenario.
2. The mp-AP MVDR produces the optimal weight vector of an antenna array for the optimal problem of the receiving beam based on a multiparametric approach [18].
3. The SVR-AS requires the radiation pattern as the train data to find the optimal weight vector of an antenna array [27].

The benefits of the proposed approach are as follows.

1. The nulling extent can be deepened.
2. This approach can guarantee that the nulling levels in the specified areas are better than conventional MVDR.
3. This approach does not require pretrain data and does not require complex mathematical computation to identify the optimal weight vector. Instead, merely the DOAs of the target user and interference sources are required for DM-AIS MVDR to determine its optimal weight vector for best radiation beamforming.

## 6. Simulation and Results

### 6.1. One User with Two Interferences

**Figure 3:**
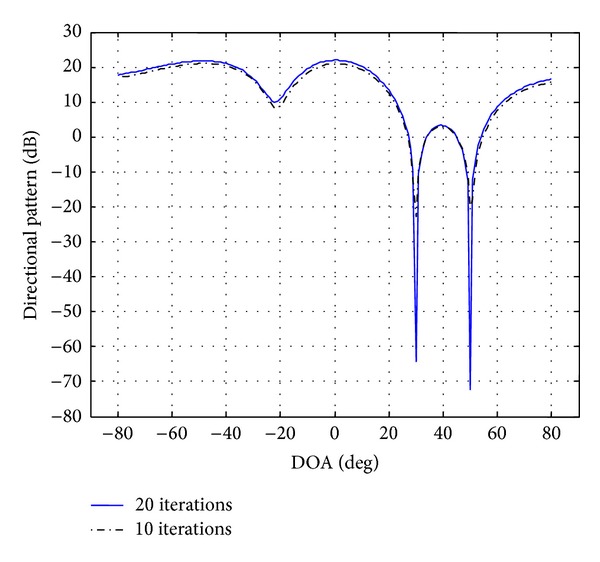
Power response DM-AIS with 10 and 20 iterations for user at 40° with interference at 30° and 50°.

In this section, simulations were conducted to validate the proposed approach. The uniform linear array (ULA) consists of four elements (*M* = 4) equispaced by half-wavelength. The desired signal and two interference signals are plane waves impinging on the ULA from the directions 50°, 30°, and 40°, respectively. In this simulation, the SNR from 5 dB to 30 dB was used for the desired signal and the two interference signals. The beam pattern nulling level should be below −70 dB. The complex vector of beamformer weights calculated by the aforementioned (27)–(31) is presented in Table 2, whereas the beam patterns generated are plotted in Figure 3. All the beam patterns have nulls at the DOAs of the interference signals and maintain a distortionless response for the SOI. However, DM-AIS place deep nulls (with nulling level equal to −80 dB) at the DOAs of two interference signal sources. The MVDR after 10 iterations reduces nulling levels compared with the MVDR after 20 iterations (Table 1).

Figure 3 gives the ratio of SINR 67.9479 dB in 20 iterations while at 10 iterations the SINR is 40.5387 dB of the aforementioned beamformer for the previous scenario. It can be seen that the DM-AIS at 20 iterations shows better ratio which is 122.49% of improvement. If we compare the improvement in SINR between normal AIs and DM-AIS for the same scenario in 10 iterations we obtain the percentage 131.48%.

**Figure 4:**
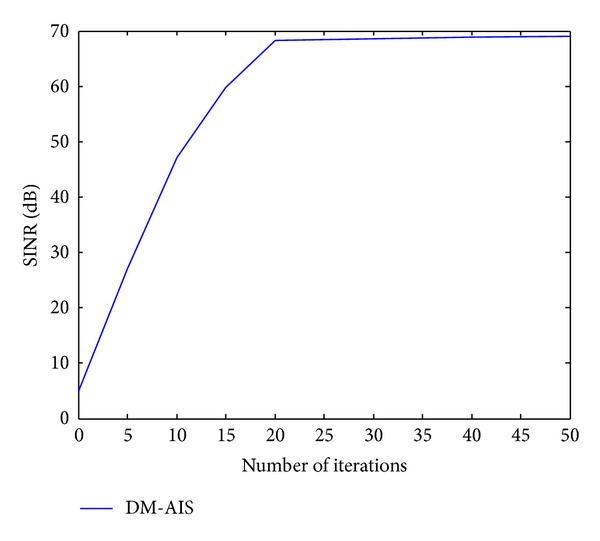
Number of iterations with SINR of DM-AIS for one user at 40° and interference at 50° and 30° by using 4 elements.

But an insignificant increase in the SINR of 0.2 is obtained for an increase in the number of iterations. The iterations were taken from 30 till 50, as shown in Figure 4. This implies that there is no need to increase the time required for simulations. The 20 iterations will give the best results by the short time. These results should be saved. The simulated results demonstrate that the DM-AIS with dynamic mutation rate give a good result effectively at a faster speed. The simulated results are dynamically adapting its value when the SINR changes. In comparison, the dynamic mutation rate has shown better results with finer resolutions.

**Figure 5:**
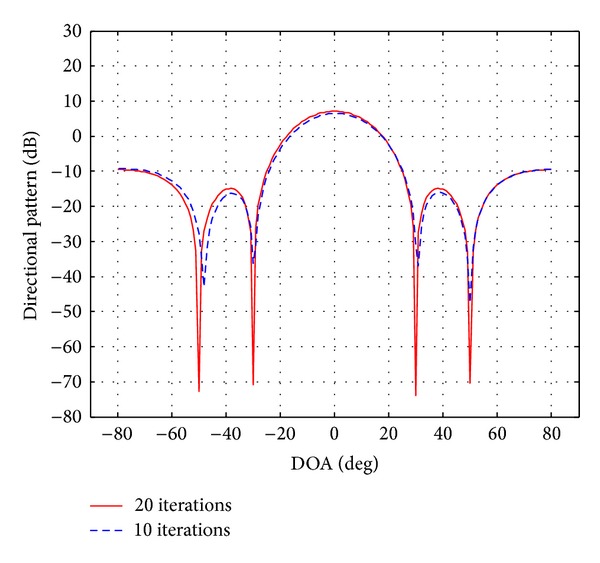
Power response for two users at 0 and 10 interference sources at 50°, −50°, 30°, and −30° with DM-AIS after 10 and 20 iterations.

We can then discriminate the difference in results after using the new method with DM-AIS. To justify the results, the new method must be proven to be more effective than any previously suggested method. The new method should have more applications by increasing the number of users and reducing interference. The results shown in Table 3 and Figure 5 provide details for two users at angles 0° and 10° with interference at angles 50°, −50°, 30°, and −30°.

### 6.2. Comparison of Conventional MVDR with MP-QP MVDR and DM-AIS

**Figure 6:**
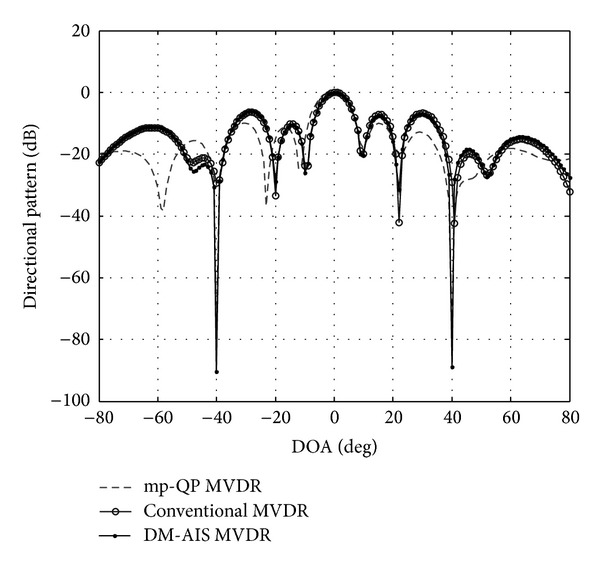
Comparison among DM-IS, mp-QP MVDR, and MVDR of one user at 0° and interference 40° and −40°.

To prove the importance of this project, the results of this work were those of previous work to enhance the MVDR in smart antennas. From the studied literature, no researcher has discussed or addressed beamforming by using four elements in the smart antenna or by applying DM-AIS in the smart antenna. Therefore, the results of this project are compared with robust MVDR beamforming for nulling level control via multiparametric quadratic programming results using 10 elements and with the direction of user at 0° with interference at 40° and −40° [8].  Figure 6 and Table 4 show the results.

Figure 6 shows that all beam patterns have nulls in the interference signals and maintain a distortionless response for the SOI. However, DM-AIS MVDR places deep nulls (with nulling level equal to −90 dB) at the two interference signal sources, whereas mp-QP MVDR obtained a null level equal to −80 dB. Therefore, the DM-AIS with MVDR response presents lower nulling levels compared with the new mp-QP MVDR. The maximum SINRs calculation for each technique is compared with one another. The maximum value obtained from DM-AIS is shown in Table 5.

This result shows that the new beamforming using artificial intelligence to determine the desired signal of user is effective and that mathematical equations or filter hardware signal processing are unnecessary. For the proposed approach, the weights value vectors make it difficult to obtain the optimum value by using any other algorithm without intensification.

## 7. Conclusion

A new DM-AIS was presented and applied in adaptive beamforming with four elements of linear antenna arrays. The proposed DM-AIS was able to enhance the MVDR technique through further optimization of weight vector, which aimed to control the nulling level of interference and the directionality of the desired signal. Very low levels of interference with good accuracy were achieved even in the case of multiple users or multiple interferences. The results of the proposed approach were compared with those of the mp-QP MVDR and conventional MVDR, and the effectiveness of the proposed approach in minimizing the power of interference and increasing SINR was observed. Finally, the DM-AIS can be useful to antenna engineers for the pattern synthesis of antenna arrays because of its good accuracy and the lack of a requirement for complicated mathematical functions.

## Figures and Tables

**Table 1:** Parameters of DM-AIS.

Dynamic mutated parameters	Value
Number of population	20
Allocated number of best population	10
Clone size	4
Number of max. iteration	20
Scaling factor	(0, 1, 2)

**Table 2:** Weight vector after 10 and 20 iterations for one user at 40° and interference at 50° and 30°.

Sensor number	Weight	10 iterations	20 iterations
1	*W*_1_	−4.5333 − 0.6902*j*	−4.6171 − 0.7212*j*
2	*W*_2_	− 2.4985 + 3.8447*j*	−2.5978 + 3.9071*j*
3	*W*_3_	−1.3543 − 1.9258*j*	−1.3966 − 1.9127*j*
4	*W*_4_	−3.7544 + 0.6641*j*	−3.7899 + 0.6865*j*

**Table 3:** Weight vector for two users with four interferences using DM-AIS.

Sensor number	Weight	10 iterations	20 iterations
1	*W*_1_	0.3330 − 0.2789*j*	0.3652 − 0.2806*j*
2	*W*_2_	0.4506 − 0.0414*j*	0.4622 − 0.0401*j*
3	*W*_3_	0.4590 + 0.0487*j*	0.5500 + 0.0474*j*
4	*W*_4_	0.3348 + 0.2723*j*	0.3292 + 0.2748*j*

**Table 4:** Weight vector values of conventional MVDR, mp-QP MVDR, and DM-AIS.

Sensor number	Conventional MVDR	mp-QP MVDR	DM-AIS MVDR
1	0.1626 − 0.0118*j*	0.1077 + 0.0469*j*	0.1594 − 0.0123*j*
2	0.1249 + 0.0093*j*	0.1269 + 0.0072*j*	0.1224 + 0.0123*j*
3	0.0630 − 0.0026*j*	0.0808 + 0.0264*j*	0.0626 − 0.0020*j*
4	0.0143 + 0.0059*j*	0.0441 − 0.0007*j*	0.0185 + 0.0055*j*
5	0.1264 + 0.0058*j*	0.1106 − 0.0188*j*	0.1196 + 0.0110*j*
6	0.1107 − 0.0291*j*	0.1043 − 0.0196*j*	0.1232 − 0.0308*j*
7	0.0212 − 0.0075*j*	0.1015 − 0.0267*j*	0.0180 − 0.0080*j*
8	0.0794 − 0.0279*j*	0.0846 − 0.0012*j*	0.0747 − 0.0209*j*
9	0.1337 + 0.0280*j*	0.1293 + 0.0069*j*	0.1274 + 0.0230*j*
10	0.1639 + 0.0306*j*	0.1102 − 0.0204*j*	0.1743 + 0.0223*j*

**Table 5:** Illustration of the SINR for DM-AIS, mp-QP MVDR, and MVDR.

MVDR SINR (dB)	mp-QP MVDR SINR (dB)	DM-AIS MVDR SINR (dB)
25	76.9897	86.9897
